# Stage-Dependent Dynamics and Assembly Processes of *PhoD*-Harboring Bacterial Communities Driven by *Ulva prolifera* Green Tides

**DOI:** 10.3390/microorganisms14071387

**Published:** 2026-06-23

**Authors:** Long Gao, Xintong Li, Rongxin Zhu, Hao Dong, Yanxue Kou, Hui He, Min Wang

**Affiliations:** 1 MoE Key Laboratory of Evolution & Marine Biodiversity, Institute of Evolution & Marine Biodiversity, Frontiers Science Center for Deep Ocean Multispheres and Earth System, College of Marine Life Sciences, Ocean University of China, Qingdao 266003, China; gaolong@stu.ouc.edu.cn (L.G.); lxt7534@163.com (X.L.); zhurongxin@stu.ouc.edu.cn (R.Z.); donghao4684@stu.ouc.edu.cn (H.D.); kouyanxue2023@163.com (Y.K.); mingwang@ouc.edu.cn (M.W.); 2 Haide College, Ocean University of China, Qingdao 266100, China; 3 UMT-OUC Joint Centre for Marine Studies, Ocean University of China, Qingdao 266003, China

**Keywords:** green tide, *phoD*-harboring bacteria, community assembly, abundant taxa, rare taxa

## Abstract

The *phoD* gene encodes alkaline phosphatase, which hydrolyzes organic phosphorus and releases bioavailable phosphorus for direct utilization by marine organisms. *phoD*-harboring bacteria are reported to be sensitive to environmental changes. As a common ecological disturbance, annual *Ulva prolifera* green tides in the southern Yellow Sea pose significant ecological challenges, yet the responses and assembly processes of *phoD*-harboring bacterial communities remain poorly understood. In this study, high-throughput sequencing was used to characterize these communities across the pre-bloom, bloom and post-bloom stages. The results revealed significant stage-specific shifts in community structure, with the bloom and post-bloom stages exhibiting higher similarity to each other than the pre-bloom stage. Abundant taxa were more sensitive to environmental fluctuations across all stages and were characterized by broader niche breadths but reduced phylogenetic diversity during the bloom. In contrast, rare taxa maintained relatively stable diversity but showed marked niche contraction. Neutral community model and βNTI analyses demonstrated that stochastic processes dominated community assembly overall. Green tide drove rare taxa toward heterogeneous selection and drift, while abundant taxa shifted toward homogeneous selection during the post-bloom stage. Co-occurrence network analysis showed increased microbial correlations during the bloom, implying a trend toward greater network stability of *phoD*-harboring bacterial communities under green tide disturbance. The lagged responses, functional redundancy and divergent ecological strategies of abundant and rare taxa may explain how green tides drive variations in microbes involved in the phosphorus cycle. These findings provide new insights into the microbial regulatory mechanisms of the nutrient cycle in coastal ecosystems affected by large-scale *U. prolifera* green tides.

## 1. Introduction

Phosphorus is an essential nutrient for algal growth and marine primary productivity. Increased anthropogenic activities have released large amounts of phosphorus into coastal regions, substantially altering local nutrient conditions [1,2,3]. Phosphorus in seawater primarily occurs as inorganic phosphorus (Pi) and organic phosphorus (Po) [4,5]. Pi is directly available to microorganisms, whereas Po represents a large phosphorus pool that can be converted into bioavailable forms via enzymatic hydrolysis [6]. Alkaline phosphatase (APase) is an extracellular enzyme mainly synthesized by microorganisms and catalyzes the hydrolysis of phosphomonoesters [7]. Bacterial APases are commonly encoded by the homologous genes *phoA*, *phoD* and *phoX*, among which *phoD* is widely distributed in natural environments and is often used as a molecular biomarker to assess the diversity and distribution of related microbial communities [8,9,10]. Using alkaline phosphatase genes (*phoD* and *phoX*) and the alkaline β-helix phosphatase gene (bpp) as biomarkers, Valdespino-Castillo et al. found marked spatiotemporal variation in the phosphorus utilization potential of microbial assemblages [5]. Luo et al. showed that soil organic amendments shaped the interactions between *phoD*- and *phoC*-harboring microbial communities, leading to complex but closer associations among these functional groups [11]. In freshwater environments, Yuan et al. reported that the abundances of *phoD*-harboring microorganisms in Lake Shijiu increased significantly in spring, and enhanced Po mineralization in sediments [10]. Saavedra et al. found that APase expression and synthesis in marine habitats were largely regulated by the heterotrophic genus *Alteromonas* and also reported distinct functional diversity patterns among four major APase families [12].

Since 2007, large-scale green tides caused by *Ulva prolifera* have occurred annually in the Yellow Sea of China, posing serious threats to coastal aquaculture, local economies and tourism [13,14]. These blooms are closely associated with increased nutrient concentrations in coastal seawater, particularly phosphorus and nitrogen [6,15]. *U. prolifera* green tides originate in the Subei Shoal, where dissolved inorganic phosphate (DIP) is the dominant phosphorus form. From late April to early May, *U. prolifera* drifts northward to the north of 35° N by ocean currents and monsoon winds. In these northern areas, dissolved organic phosphate (DOP) becomes the main phosphorus form and can be hydrolyzed into bioavailable DIP by APase to sustain the development of green tides [6,16]. Previous studies have shown that *U. prolifera* green tides can induce significant changes in coastal microbial communities [17]. The abundance of culturable bacteria, especially Vibrio species, increased, and distinct structural differences between epiphytic and free-living bacterial communities have been observed [18]. During the decline stage, Liu et al. demonstrated that algal decomposition strongly regulated carbon and nitrogen cycles in the surrounding microenvironment by reshaping the structure and function of phycospheric microbial communities [19]. Despite extensive research on the effects of *U. prolifera* green tides on microbial communities, their effects on *phoD*-harboring bacterial communities remain poorly understood.

Microbial communities generally consist of abundant and rare taxa [20]. Abundant taxa typically exhibit high relative abundance but low richness and play important roles in biomass production and core ecological processes. In contrast, rare taxa have low relative abundance but high richness and may serve as a dynamic “seed bank” to support nutrient cycles and ecological stability [21]. Different microbial ecotypes often adopt distinct strategies in response to environmental change. Mo et al. reported significant differences in diversity between abundant and rare taxa in three subtropical bays [22]. Environmental selection and neutral processes were found to jointly shape similar biogeographic patterns in both groups, although rare taxa may be governed by more complex community assembly mechanisms [22]. Given that *U. prolifera* green tides strongly affect nutrient availability and microbial community structure, we hypothesize that different *phoD*-harboring bacterial ecotypes respond differently to such disturbances. This study aimed to: (i) characterize changes in community structure and assembly processes of different *phoD*-harboring bacterial ecotypes in response to *U. prolifera* green tide, and (ii) clarify how the green tide affects interactions within *phoD*-harboring bacterial communities. Our findings provide new insights into microbial adaptation mechanisms to the outbreak of *U. prolifera* green tides.

## 2. Materials and Methods

### 2.1. Samples Collection and Environmental Parameters Determination

**Figure 1:**
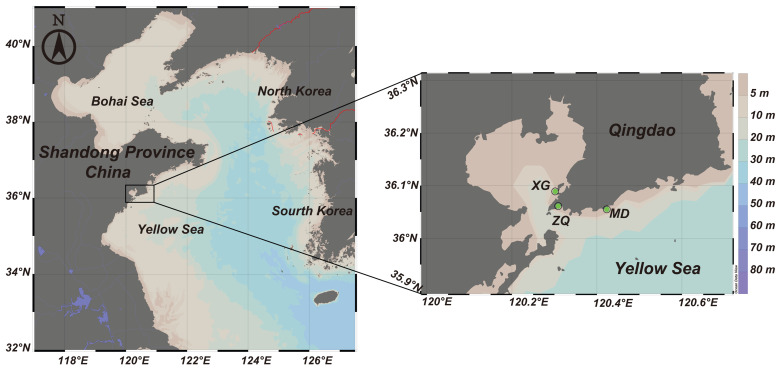
Locations of sampling stations in the *U. prolifera* green tide outbreak area along the Qingdao coast.

Surface seawater samples were collected at three stations along the Qingdao coast, including Xiaogang (XG, 36.07° N, 120.30° E), Zhanqiao Pier (ZQ, 36.06° N, 120.31° E) and Maidao (MD, 36.05° N, 120.42° E) (Figure 1). All stations were located within the area affected by *U. prolifera* green tides. Sampling was conducted from March to November 2021 twice per month from June to August and once per month during the other months. Different green tide stages were classified based on Chlorophyll *a* (Chl *a*) concentrations and field visual observations. A total of 36 samples were collected and divided into three groups: pre-bloom stage (from 24 March to 20 May), bloom stage (from 10 June to 6 August) and post-bloom stage (from 18 August to 24 November). For each sample, 2 L of seawater was sequentially filtered through an 800-mesh plankton net and a 0.22-μm pore-size polycarbonate membrane (47 mm diameter; Millipore, Burlington, MA, USA). Membranes were immediately stored at −80 °C until DNA extraction.

Environmental parameters, including temperature, salinity, dissolved oxygen (DO) concentration and pH, were measured in situ using a YSI ProPlus multi-parameter analyzer (YSI, Yellow Springs, OH, USA). Chl *a* concentration was determined by spectrophotometry after acetone extraction [23,24]. For nutrient analysis, seawater samples were filtered through 0.45-μm membranes, and the concentrations of nitrate (NO_3_^−^), nitrite (NO_2_^−^), ammonium (NH_4_^+^) and phosphate (PO_4_^3−^) were measured using a QuAAtro automated nutrient analyzer (Seal Analytical Ltd., Wrexham, UK) (Table S1).

### 2.2. DNA Extraction, PCR Amplification and High-Throughput Sequencing Analysis

Total genomic DNA was extracted using the ALFA-SEQ Magnetic Water DNA Kit (Findrop, Guangzhou, China) according to the manufacturer’s instructions. DNA quality was assessed with a NanoDrop One spectrophotometer (Thermo Fisher Scientific, Waltham, MA, USA). The phoD gene was amplified using the specific primer pair phoD-F730 (5′-TGG GAY GAT CAY GAR GT-3′) and phoD-R1083 (5′-CTG SGC SAK SAC RTT CCA-3′) [8]. PCR amplification was performed under the following conditions: initial denaturation at 94 °C for 5 min; 30 cycles of denaturation at 94 °C for 30 s, annealing at 52 °C for 30 s and extension at 72 °C for 30 s; followed by a final extension at 72 °C for 10 min. PCR products were verified by 1.5% agarose gel electrophoresis and purified using the E.Z.N.A.^®^ Gel Extraction Kit (Omega Bio-Tek, Norcross, GA, USA). High-throughput sequencing of the *phoD* gene was conducted on an Illumina NovaSeq 6000 platform by Magigene Biotechnology Co., Ltd. (Guangzhou, China).

### 2.3. Bioinformatic Processing

Raw reads were processed using QIIME2 (version 2021.4.0) with minor modifications [25]. Briefly, raw reads were imported via the q2-import plugin. Quality control, including denoising, primer trimming and chimera removal, was performed using the Deblur algorithm. High-quality reads were merged with the vsearch plugin. Referring to methods described in previous studies, operational taxonomic units (OTUs) were clustered based on a 97% sequence similarity threshold using the same plugin [26,27]. To minimize the influence of sequencing errors, singleton and doubleton OTUs were removed from subsequent analyses. The feature table was normalized using the q2-feature-table module. In accordance with previous studies, OTUs with an average relative abundance greater than 0.1% were classified as abundant taxa, whereas OTUs with an average relative abundance less than 0.002% were defined as rare taxa in this study (Figure S1) [20,22,28].

The Shannon–Wiener diversity index and Faith’s phylogenetic diversity index were calculated to evaluate alpha diversity. Beta diversity was analyzed in R (version 4.4.3) based on Bray–Curtis dissimilarity calculated using the “microtable” package (version 1.14.0) and beta mean nearest taxon distance (βMNTD) calculated using the “NST” package (version 3.1.10) [29,30]. Differences among groups were tested using Dunn’s test followed by Benjamini–Hochberg (BH) correction. Community composition was visualized with the “ggplot2” package (version 4.0.1). Community distribution patterns of abundant and rare taxa were further examined via non-metric multidimensional scaling (NMDS) analysis, and differences among stages were tested with ADONIS by "vegan" package (version 2.6-10). Representative sequences obtained in this study and reference sequences retrieved from the NCBI non-redundant database were dereplicated at a 97% sequence similarity threshold using CD-HIT (version 4.8.1). The resulting non-redundant sequences were aligned with MAFFT (version 7.525) under default parameters. A maximum-likelihood phylogenetic tree was then constructed using IQ-TREE (version 3.0.1), with branch support assessed by 5000 bootstrap replicates [31]. The phylogenetic tree annotated with OTU relative abundance and taxonomic information was visualized using iTOL (Interactive Tree Of Life, version 7.5) [32]. Mantel tests were performed to examine the relationship between environmental factors and β-diversity based on Bray–Curtis dissimilarity. The effects of environmental factors on community structure were further assessed using distance-based redundancy analysis (dbRDA).

### 2.4. Assembly Processes Analysis

The assembly processes of abundant and rare taxa were analyzed using a null model framework [29,33]. The beta nearest taxon index (βNTI) was calculated by comparing the observed βMNTD with the mean of the null distribution. |βNTI| > 2 indicates that community turnover is primarily governed by deterministic processes, whereas |βNTI| ≤ 2 suggests a dominant influence of stochastic processes. The “iCAMP” package (v1.5.12) was further used to identify the specific stochastic assembly processes [34]. Specifically, a modified Raup–Crick metric based on Bray–Curtis distance (RCbray-curtis) was calculated. RCbray-curtis > 0.95 indicates dispersal limitation, RCbray-curtis < −0.95 implies homogeneous dispersal, and |RCbray-curtis| < 0.95 indicates ecological drift.

### 2.5. Co-Occurrence Network Analysis

Co-occurrence networks of *phoD*-harboring bacterial communities were constructed using the “igraph” package in R (version 4.4.3). Only OTUs with a relative abundance greater than 0.005% and presence in at least half of the samples were retained. We first computed pairwise Spearman correlations for the filtered OTU dataset. The Benjamini–Hochberg FDR approach was applied to correct multiple testing biases. Correlations with corrected *p* < 0.05 and |*r*| > 0.6 were finally retained as significant associations [35,36]. Networks were visualized with Gephi (version 0.10.1), and key topological properties, including average degree, average clustering coefficient and modularity, were calculated for each network [37]. To evaluate the stability of *phoD*-harboring bacterial communities under the influence of *U. prolifera* green tide, network robustness and vulnerability were analyzed according to the methods described by Yuan et al. [38].

## 3. Results

### 3.1. Diversity and Composition of Different PhoD-Harboring Bacterial Ecotypes

**Figure 2:**
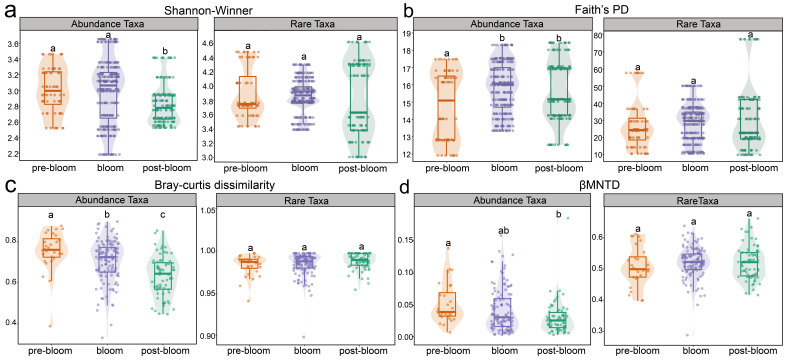
Shannon–Wiener diversity index (**a**), Faith’s phylogenetic diversity index (**b**), Bray–Curtis dissimilarity (**c**) and βMNTD (**d**) of different phoD-harboring bacterial ecotypes in pre-bloom, bloom and post-bloom stages. Different lowercase letters indicate significant differences among groups (*p* < 0.05).

**Figure 3:**
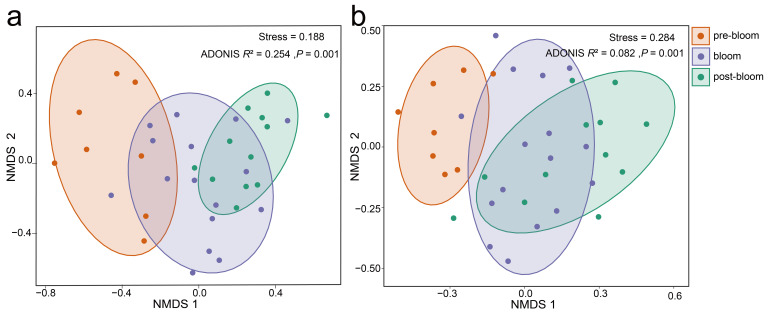
NMDS analysis of abundance (**a**) and rare taxa (**b**) in pre-bloom, bloom and post-bloom stages.

A total of 4760 OTUs were obtained after quality control, and the three periods exhibited distinct OTU counts (Figure S2). Alpha diversity, represented by the Shannon–Wiener diversity index and Faith’s phylogenetic diversity index, exhibited distinct patterns between abundant and rare taxa. For abundant taxa, taxonomic diversity increased slightly from the pre-bloom stage to the bloom stage but decreased significantly during the post-bloom stage, peaking during bloom and falling to its lowest level after green tide recession. Phylogenetic diversity of abundant taxa differed notably between the bloom and post-bloom stages (*p* < 0.05; Figure 2a), with markedly lower values during the post-bloom stage than bloom stage. In contrast, both taxonomic and phylogenetic diversity of rare taxa fluctuated slightly across the three stages (Figure 2b), with no clear maximum or minimum observed. Beta diversity also showed distinct patterns between abundant and rare taxa. For abundant taxa, spatial variability decreased progressively across the three stages (*p* < 0.05; Figure 2c), indicating that the green tide gradually reduced community dissimilarity; accordingly, abundant bacterial community exhibited the highest dissimilarity during the pre-bloom stage and the lowest during the post-bloom stage. However, the spatial variability of rare taxa remained relatively stable (Figure 2d). Notably, phylogenetic turnover among rare taxa increased slightly under the influence of green tide, reaching its maximum during the post-bloom stage. NMDS analysis further revealed that the community composition of abundant taxa varied significantly among all three stages, whereas rare taxa exhibited significant differences only between the pre-bloom and bloom stages and between the pre-bloom and post-bloom stages (*p* < 0.01; Figure 3a,b).

### 3.2. Phylogenetic Characteristics of Different phoD-Harboring Bacterial Ecotypes

**Figure 4:**
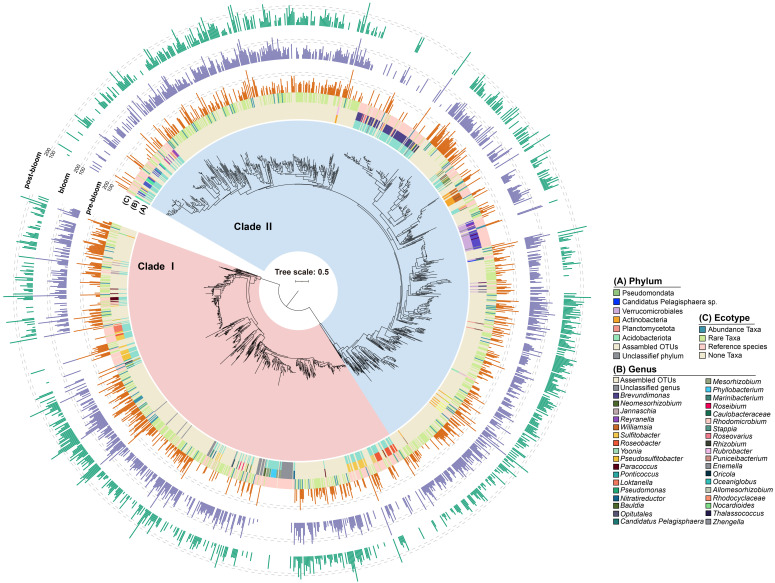
Phylogenetic analysis of *phoD*-harboring bacterial communities. Panels A and B show the taxonomic composition at the phylum and genus levels, respectively. In panel C, pink indicates reference sequences, green indicates rare taxa, blue indicates abundant taxa, and yellow indicates microbial sequences assembled in this study. The outermost concentric tracks, ordered from inner to outer, display the abundance profiles of assembled OTUs across the pre-bloom, bloom and post-bloom stages, respectively. Dashed lines mark the relative abundance thresholds of 100 and 200.

To characterize the phylogenetic relationships and taxonomic distribution of *phoD*-harboring bacteria, a circular phylogenetic tree that contained 1460 branches was constructed (Figure 4). The *phoD*-harboring bacterial community was clearly divided into two monophyletic clades, indicating significant evolutionary divergence among these taxa. At the phylum level, Clade I was primarily composed of Proteobacteria, whereas Clade II exhibited broader phylogenetic diversity, including Verrucomicrobiota, Actinobacteriota and Acidobacteriota in addition to Proteobacteria (Figure 4, Panel A). At the genus level, Clade I was dominated by *Sulfitobacter*, *Roseobacter*, *Loktanella* and *Phyllobacterium* (Figure 4, Panel B). In contrast, Clade II contained a high proportion of uncultured lineages, such as uncultured *Akkermansiaceae* and uncultured *Verrucomicrobiales*, highlighting the widespread presence of uncultured taxa within this clade (Figure 4, Panel B). Relative abundance differed markedly between the two clades (Figure 4). Clade I contained most of the high abundant OTUs and was dominated by abundant taxa, including *Rhizobium*, *Erythrobacter* and *Marinococcus*. Clade II was mainly composed of OTUs with low to moderate abundance, with rare taxa accounting for a relatively high proportion. The distinct distribution patterns of abundant and rare taxa between the two clades indicated their potential niche differentiation under the influence of *U. prolifera* green tide (Figure S3). These patterns also reflected that abundant taxa had a relatively simple community composition, whereas rare taxa displayed higher species richness with lower relative abundance.

### 3.3. Effects of Environmental Parameters on Different PhoD-Harboring Bacterial Ecotypes

**Figure 5:**
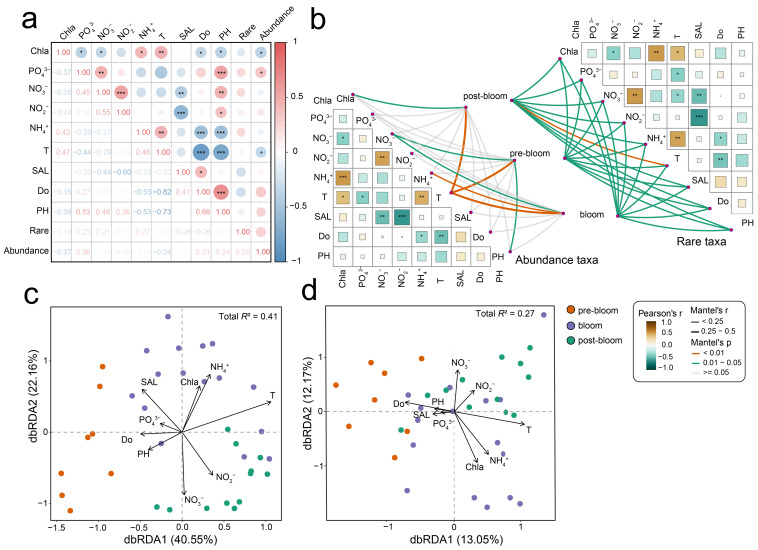
Correlation analysis between environmental factors and different *phoD*-harboring bacterial ecotypes. (**a**) Pearson correlation heatmap between environmental factors and alpha diversity indices of abundant and rare taxa; red indicates positive correlations, blue indicates negative correlations, and color gradient corresponds to correlation degree. (**b**) Mantel test between environmental parameters and community composition of abundant and rare taxa; line thickness corresponds to correlation strength. (**c**) dbRDA ordination of community distribution patterns of abundant taxa, with symbols colored by stage. (**d**) dbRDA ordination of community distribution patterns of rare taxa, with symbols colored by stage. Asterisks indicate statistically significant differences: * *p* < 0.05, ** *p* < 0.01, *** *p* < 0.001.

Environmental factors exhibited pronounced variations among the three periods (Figure S4). Spearman correlation analysis revealed that the alpha diversity of abundant taxa was significantly negatively correlated with Chl *a* concentration and temperature, but significantly positively correlated with PO_4_^3−^ concentration (*p* < 0.05). Alpha diversity of rare taxa was positively correlated with PO_4_^3−^ concentration, NO_3_^−^ concentration, NO_2_^−^ concentration and NH_4_^+^ concentration, and negatively correlated with DO concentration and salinity; however, none of these correlations were statistically significant (*p* > 0.05). Overall, although some correlations between alpha diversity indices and environmental factors were observed, most were not statistically significant (*p* > 0.05; Figure 5a, Table S2). The Mantel test further clarified the relationships between community composition and environmental factors across the three stages (Figure 5b). Before the bloom, abundant taxa were significantly correlated with temperature (*p* < 0.01), NO_3_^−^ concentration (*p* < 0.01) and pH (*p* < 0.01). During the *U. prolifera* green tide, abundant taxa were significantly correlated with NH_4_^+^ concentration (*p* < 0.01) and temperature (*p* < 0.01). After the bloom, the strength of these correlations between abundant taxa and environmental factors decreased markedly. In contrast, rare taxa exhibited significant correlations with environmental factors across all three stages (*p* < 0.05). To further evaluate the influence of environmental factors on each ecotype, distance-based redundancy analysis (dbRDA) based on Bray–Curtis dissimilarity was performed. For abundant taxa, the model explained a relatively large proportion of community variation (*R*^2^ = 0.41). Temperature (*p* < 0.001) and NH_4_^+^ concentration (*p* < 0.001) were the strongest explanatory variables, followed by salinity (*p* < 0.01), NO_3_^−^ concentration (*p* < 0.01), NO_2_^−^ concentration (*p* < 0.05), DO concentration (*p* < 0.05) and pH (*p* < 0.05; Figure 5c). For rare taxa, the variance explained by the model was lower (*R*^2^ = 0.27), with Chl *a* concentration (*p* < 0.01), NO_3_^−^ concentration (*p* < 0.01) and temperature (*p* < 0.01) identified as significant drivers of community variation (Figure 5d).

### 3.4. Phylogenetic Diversity and Assembly Processes of Different PhoD-Harboring Bacterial Ecotypes

**Figure 6:**
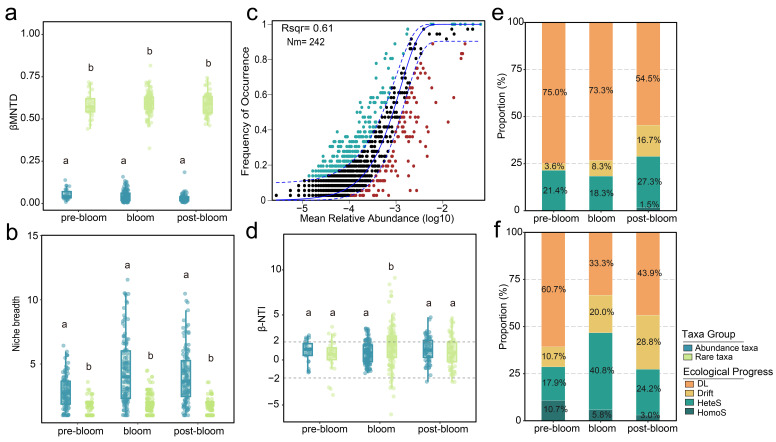
Phylogenetic diversity, niche breadth and community assembly processes of different *phoD*-harboring bacterial ecotypes. (**a**) βMNTD values of abundant and rare taxa across three stages. (**b**) Niche breadth of abundant and rare taxa across three stages. (**c**) Fit of the neutral community model for *phoD*-harboring bacterial communities. (**d**) Distribution of βNTI of abundant and rare taxa. (**e**) Relative contributions of deterministic and stochastic processes to the assembly of abundant taxa. (**f**) Relative contributions of deterministic and stochastic processes to the assembly of rare taxa. Different lowercase letters indicate significant differences among groups (*p* < 0.05).

Phylogenetic diversity, measured as βMNTD, varied significantly across the three stages for abundant taxa (*p* < 0.05) but not for rare taxa (*p* > 0.05; Figure 2d and Figure 6a). Both ecotypes exhibited the highest phylogenetic diversity during the bloom stage (Figure 6a). Abundant taxa showed a broader niche breadth than rare taxa. Niche breadth peaked during the bloom stage for both ecotypes; however, it remained relatively broad for abundant taxa in the post-bloom stage, whereas for rare taxa it decreased to a level comparable to that observed in the pre-bloom stage (Figure 6b and Figure S5).

The neutral community model (NCM) indicated that stochastic processes played an important role in the assembly of *phoD*-harboring bacterial communities (*R*^2^ = 0.61; Figure 6c and Figure S5). βNTI analysis further confirmed that stochastic processes primarily governed the assembly of both abundant and rare taxa (Figure 6d and Figure S5). To quantify the relative contributions of deterministic and stochastic processes, we used a combined approach based on βNTI and the modified Raup–Crick metric [29,39]. The results showed that stochastic processes predominated in both ecotypes across the three stages, accounting for more than 55% of the community assembly processes (Figure 6e,f). For abundant taxa, community assembly was mainly driven by dispersal limitation; however, the relative contributions of ecological drift and heterogeneous selection increased notably in the post-bloom stage, suggesting adaptive responses to environmental disturbances (Figure 6e). In contrast, although stochastic processes generally dominated the assembly of rare taxa, heterogeneous selection became the primary driver during the bloom stage, whereas dispersal limitation and ecological drift were more important during the pre-bloom and post-bloom stages (Figure 6f). These results indicate that abundant and rare taxa adopted distinct assembly strategies under the influence of *U. prolifera* green tide. Abundant taxa appeared to maintain community stability mainly through dominant dispersal limitation combined with moderate environmental filtering, whereas rare taxa showed greater uncertainty, likely due to stronger ecological drift and fluctuations in environmental filtering.

### 3.5. Coexistence Patterns and Network Stability of PhoD-Harboring Bacterial Communities

**Figure 7:**
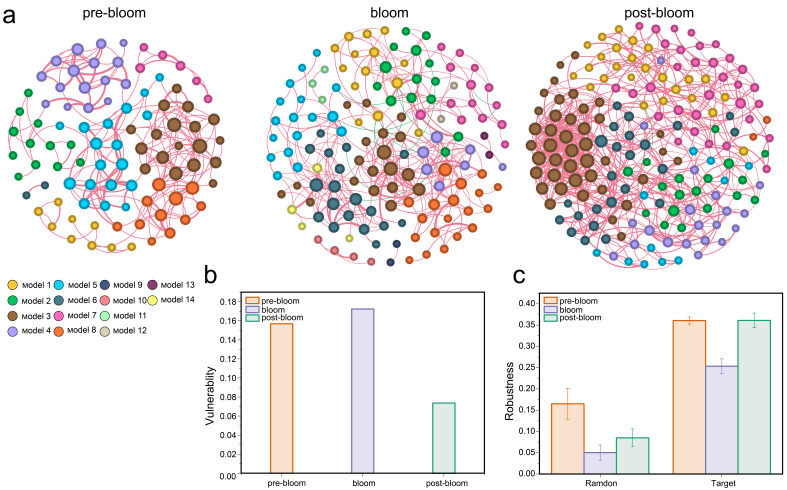
Co-occurrence patterns and network stability of *phoD*-harboring bacterial communities. (**a**) Co-occurrence network analysis of *phoD*-harboring bacterial communities across three stages. (**b**) Vulnerability analysis of *phoD*-harboring bacterial networks across three stages. (**c**) Robustness analysis of *phoD*-harboring bacterial networks under random and targeted node removal across three stages. Random robustness was evaluated by random removal of 50% nodes in each network, and targeted robustness was assessed by removal of five key nodes in each network. Standard deviations were calculated based on 100 simulations.

To investigate the coexistence patterns of *phoD*-harboring bacterial communities under the influence of *U. prolifera* green tide, co-occurrence networks were constructed for each of the three stages. Distinct differences in network topology were observed among stages (Figure 7a). The post-bloom network contained 173 nodes and 667 edges, showing greater complexity than the pre-bloom and bloom networks, as further supported by the highest average degree observed in the post-bloom network (4.674, 4.921 and 7.711, respectively). The average clustering coefficient decreased over time (0.506, 0.413 and 0.408), suggesting stronger local aggregation in the pre-bloom stage which may facilitate the formation of stable small-scale assemblages at this stage. The average path length also decreased across the three stages (5.476, 4.213 and 3.870), indicating a gradual increase in network connectivity efficiency over time. The bloom network had the largest number of modules and the highest modularity (0.643, 0.721 and 0.574), implying the division of more independent functional submodules during this stage.

Network stability of *phoD*-harboring bacterial communities also varied across stages. Higher vulnerability observed in the bloom stage indicated that the *phoD*-harboring bacterial network was more sensitive to disturbances at this stage (Figure 7b). Meanwhile, network robustness was lower during bloom stage than pre-bloom and after-bloom stages, suggesting reduced community resilience in response to the outbreak of *U. prolifera* green tide (Figure 7c).

## 4. Discussion

Phosphorus is a critical limiting nutrient in marine ecosystems. The DOP in seawater is predominantly composed of phosphate esters, whose hydrolysis is jointly catalyzed by cell-bound extracellular enzymes and cell-free dissolved extracellular enzymes, among which APases serve as core functional enzymes that drive marine DOP cycling and phosphorus biogeochemical turnover [12].

The outbreak of *U. prolifera* green tides reshaped the diversity of *phoD*-harboring bacterial communities through environmental stress and potential allelopathic effects [40]. Alpha diversity analysis revealed that core populations benefited from the bloom, as their diversity increased from the pre-bloom to the bloom stage but decreased significantly in the post-bloom stage (*p* < 0.05). By contrast, rare taxa members followed an opposite trend, with Shannon index declining during the bloom and rising afterwards. Furthermore, both community dissimilarity and phylogenetic turnover of dominant groups decreased continuously across the entire succession (*p* < 0.05). In contrast, rare taxa maintained relatively stable phylogenetic diversity, indicating stronger resistance to environmental disturbances [22,41]. The response pattern of abundant taxa may be attributed to their dominance during the bloom, which drove the community toward homogenization and subsequently reduced beta diversity. This finding is consistent with previous studies on seasonal variations in microbial communities influenced by green tides in coastal waters of Jiangsu Province [42]. During the post-bloom stage, the decomposition of *U. prolifera* released organic phosphorus, which may stimulate microbial alkaline phosphatase activity and shift the dominant community function from proliferation to organic matter degradation. As the dominance of abundance taxa decreased in the post-bloom stage, rare taxa that act as a microbial “seed bank” expanded their ecological niches, and their community composition became more diverse, leading to a slight increase in beta diversity [21]. In this study, correlation analysis indicated that the alpha diversity of abundant taxa was significantly positively correlated with DO concentration, pH and salinity, suggesting a preference for stable and aerobic marine environments. In contrast, rare taxa were positively correlated with NO_3_^−^ and NH_4_^+^ concentrations but negatively correlated with DO concentration. This pattern suggests that rare taxa may avoid competition with abundant taxa by occupying distinct nutritional niches within micro-anoxic microenvironments in algal aggregates. Higher Chl *a* concentrations tended to inhibit both abundant and rare taxa. A clear negative correlation was observed for abundant taxa, indicating that this group may be more sensitive to green tide outbreaks. Temperature and NH_4_^+^ concentration were also key drivers of variations in abundant taxa community across stages. Although environmental factors also acted on rare taxa, no predominant factor was identified in this study.

Our study revealed significant phylogenetic differentiation within *phoD*-harboring bacterial communities, which formed two independent monophyletic clades. This differentiation suggested that *phoD*-harboring bacteria may possess distinct evolutionary and ecological strategies for adapting to environmental changes induced by *U. prolifera* green tides. Clade I contained a relatively high proportion of abundant taxa, mainly affiliated with Proteobacteria including representative genera *Thiobacillus* and *Roseobacter*. These taxa are widely recognized as important contributors to the coastal phosphorus cycle [8,43], and their high efficiency in utilizing labile substrates released during the proliferation and decomposition of *U. prolifera* suggest that they may act as major drivers of organic phosphorus mineralization [40,44]. In contrast, Clade II comprised a broader phylogenetic lineage and contained a higher proportion of rare taxa, including members of Verrucomicrobia and Actinobacteria, as well as many uncultured lineages. These rare taxa may contribute to the degradation of complex organic phosphorus compounds, which complements the role of abundant taxa in Clade I. The enrichment of diverse taxa in Clade II further indicated that environmental fluctuations induced by green tides may promote niche differentiation among *phoD*-harboring bacteria. Such niche differentiation could reduce interspecific competition and enhance functional redundancy, thereby improving the stability of *phoD*-harboring bacterial communities under the influence of *U. prolifera* green tides.

Regarding niche characteristics and community structuring processes, dominant populations within the *phoD*-harboring bacterial community exhibited wider niche breadth and stronger temporal dynamics during the bloom phase [45,46]. This trait may have contributed to their capacity to utilize diverse organic phosphorus substrates. Notably, these microbes maintained a relatively high niche breadth even in the post-bloom stage, which may indicate their potential ability to utilize residual organic matter and continuous contribution to drive phosphorus mineralization. Stochastic processes dominated the assembly of abundant taxa across all three stages. During the bloom stage, increased environmental homogeneity slightly reduced the contribution of deterministic processes and enhanced the relative importance of ecological drift, thereby contributing to reduced beta diversity. Following bloom decline, the massive decomposition of *U. prolifera* residues increased the proportions of both ecological drift and deterministic processes, particularly heterogeneous selection, which further influenced community turnover [47]. By comparison, rare taxa members possessed narrower niche breadth yet followed similar temporal variation patterns. During the bloom stage, deterministic forces exerted stronger effects on this fraction, though stochasticity still played a considerable role. This pattern is likely linked to their small population sizes and strict habitat specialization [27,48], and aligns with prior findings that fluctuating nutrient availability favors distinct alkaline phosphatase variants [39,49]. Throughout the bloom stage, the adaptive niche dynamics of abundance taxa and the disturbance resistance of rare taxa jointly may imply the stability of the phosphorus cycle. Abundant taxa may have relieved nutrient limitation via continuous organic phosphorus mineralization, while rare taxa preserved functional redundancy. This complementary mechanism may serve as an important guarantee for sustaining the phosphorus cycle in response to environmental disturbances [20,50].

We further constructed co-occurrence networks using all filtered OTUs, which included both abundant and rare taxa. Microbial correlational patterns are important factors that correlate with community compositions [51]. The results showed that the *phoD*-harboring bacterial network exhibited a gradual expansion from the pre-bloom stage to the post-bloom stage, as indicated by increases in the number of nodes, edges and average degree. During the bloom stage, the rapid proliferation of *U. prolifera* likely released more phosphorus-containing metabolites, which potentially strengthened statistical associations within the *phoD*-harboring bacterial community. Under environmental filtering, the community tended to form more specialized functional modules rather than extensive connections across the whole network, a pattern accompanied by increased modularity and a reduced average clustering coefficient [52]. This modular organization may improve functional specialization but may also reduce overall network integration, which could correspond to higher vulnerability and lower robustness during the bloom stage [53,54]. This pattern is consistent with the observation that the alpha diversity of dominant members was relatively high during this phase and declined afterward. In the post-bloom stage, the decomposition of *U. prolifera* created a more complex and heterogeneous aquatic environment, which likely required stronger synergistic interactions among various phosphorus cycle functional groups [4]. Higher diversity of low-abundance microbes, greater species richness and fragmented habitats jointly may have collectively contributed to network expansion and an increase in average degree. Reduced clustering coefficient and modularity suggested weaker compartmentalization and more extensive cross-group correlations. Consequently, the network tended to display the lowest vulnerability, robustness and improved resistance to environmental disturbance [55]. Rare taxa, acting as a microbial seed bank, may provide potential nodes and reduce the risk of network collapse. This pattern reflects a functional shift of the *phoD*-harboring bacterial community from a green tide-specialized state during the bloom stage to a broader environmental adaptation strategy in the post-bloom stage. These correlational changes in network connectivity may favor material exchange and signal transmission among taxa, potentially improving the overall phosphorus mineralization capacity of the community [56,57]. Future studies should incorporate a larger number of sampling stations across a broader spatial gradient to confirm the generality of our findings. It is necessary to acknowledge the inherent methodological limitations of co-occurrence network inference. These networks only reflect statistical correlations rather than authentic biological interactions. Furthermore, severe data zero inflation caused by the inclusion of rare taxa, coupled with multiple pairwise testing, inevitably introduces potential false-positive correlations.

The current study is limited to sequencing-based correlation inference without functional validation of the phosphorus cycle, and lacks quantitative data on *Ulva prolifera* biomass, coverage and remote sensing, which may restrict the interpretation of bloom intensity–microbial dynamics relationships. Future studies will expand spatial sampling to verify result generality, and integrate in situ measurements with remote sensing to establish more robust quantitative linkages.

## 5. Conclusions

Overall, abundant and rare *phoD*-harboring bacterial taxa display distinct ecological strategies during the succession of *Ulva prolifera* green tides. Abundant taxa with wider niche breadths are primarily structured by stochastic assembly processes and exhibit higher sensitivity to disturbances; their diversity peaks at the bloom stage, coupled with progressive community homogenization, and they may contribute to sustained organic phosphorus mineralization. In contrast, rare taxa characterized by narrower, specialized niches are more strongly shaped by deterministic processes during the bloom phase. They maintain stable phylogenetic diversity and greater disturbance resistance, and may function as a microbial “seed bank” to preserve functional redundancy within the community. The *phoD*-harboring bacterial community was divided into two clades. Clade I was dominated by abundant taxa, which may maintain the basic efficiency of phosphorus mineralization; Clade II comprised rare taxa and uncultured lineages, which may contribute to the degradation of complex organic phosphorus. During the bloom, increased environmental homogeneity was associated with a relatively highly modular network that may have exhibited weaker disturbance resistance. In the post-bloom stage, algal decomposition may have increased environmental heterogeneity, which correlated with the apparent restoration of network robustness and a potential trend toward broader environmental adaptation of the community. These findings provide new insights into the dynamics of *phoD*-harboring communities under *U. prolifera* green tide events in coastal Qingdao areas.

## Data Availability

The raw reads were submitted to National Centre for Biotechnology Information (NCBI) under the accession number PRJNA1464139. (http://www.ncbi.nlm.nih.gov/bioproject/1464139, accessed on 10 May 2026).

## Supplementary Materials

The following supporting information can be downloaded at: https://www.mdpi.com/article/10.3390/microorganisms14071387/s1, Figure S1: Median of relative abundance for individual OTUs; Figure S2. (a) Venn diagram of OTU numbers across three periods; (b) Clustered bar chart of sampling stations and dominant OTUs (relative abundance > 0.1%); Figure S3. (a) Venn diagram of OTU numbers of abundant taxa across three periods; (b) Venn diagram of OTU numbers of rare taxa across three periods; Figure S4. Box plot of environmental factor differences among three periods. (a) Chlorophyll *a* (Chl *a*); (b) Temperature (T); (c) Salinity; (d) Dissolved oxygen (DO); (e) PH; (f) ammonium (NH_4_^+^); (g) nitrite (NO_2_^−^); (h) nitrate (NO_3_^−^); (i) phosphate (PO_4_^3−^); Figure S5. (a) Niche width and βNTI index of abundant taxa; (b) Niche width and βNTI index of rare taxa; (c) Neutral model analysis across three periods. Values with different lowercase letters are significantly different (*p* < 0.05); Table S1: Detailed information on the 39 samples; Table S2: Spearman correlations among environmental factors and with Shannon diversity of abundant and rare taxa.
