# Supplementary material for: Stage-Dependent Dynamics and Assembly Processes of PhoD-Harboring Bacterial Communities Driven by Ulva prolifera Green Tides
Source: Microorganisms. 2026 Jun 23;14(7):1387. doi: 10.3390/microorganisms14071387 (PMC13413677; doi:10.3390/microorganisms14071387)
Supplement: Supplementary file 1 [file microorganisms-14-01387-s001.zip › Supplyment figures.pdf]

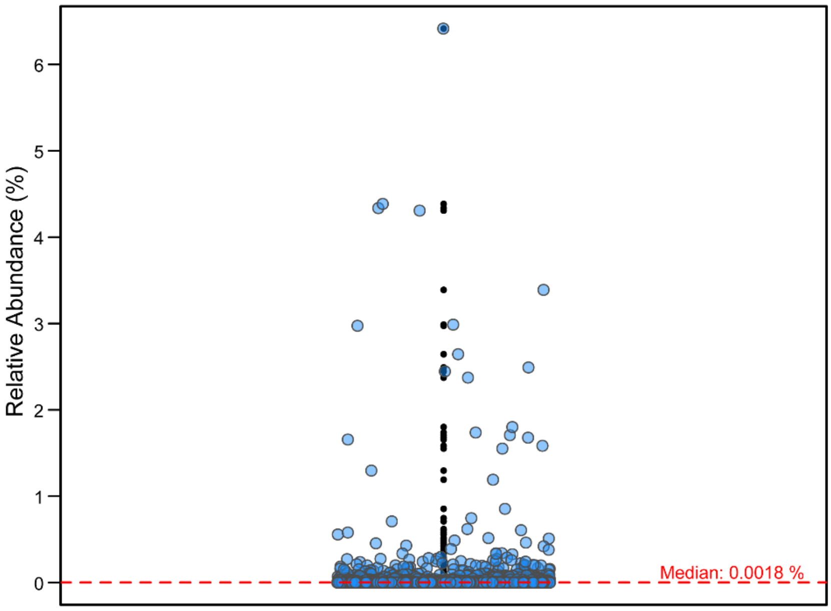

Figure S1. Median of relative abundance for individual OTUs

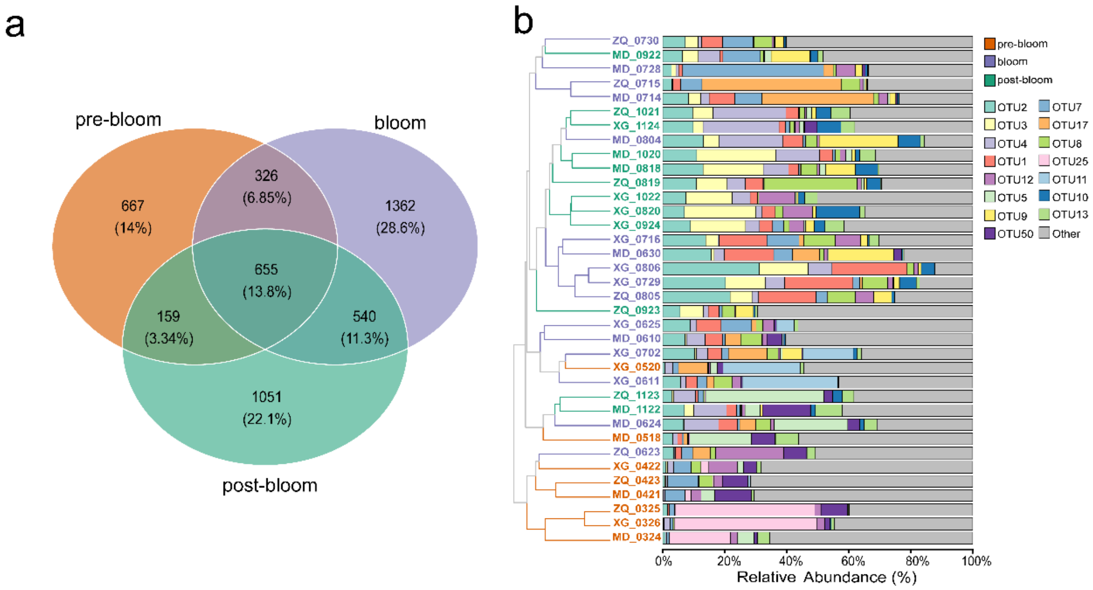

Figure S2. (a) Venn diagram of OTU numbers across three periods

(b) Clustered bar chart of sampling stations and dominant OTUs (relative abundance > 0.1%)

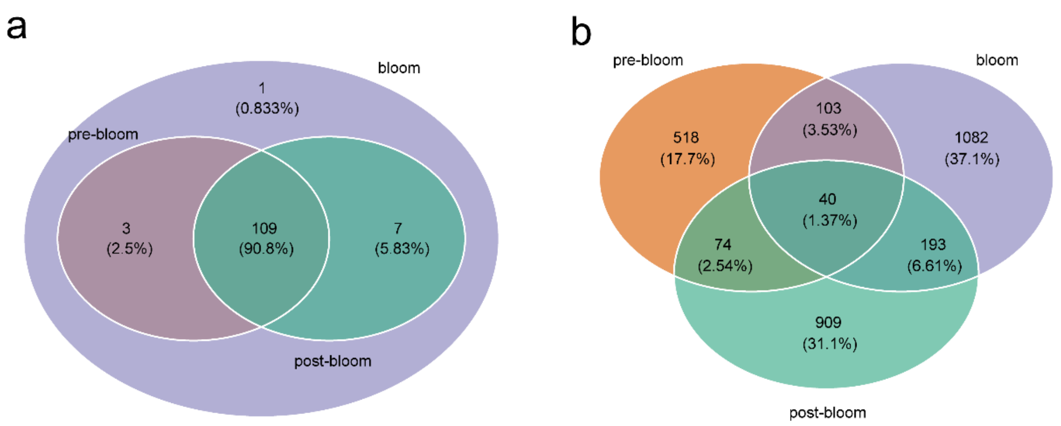

Figure S3. (a) Venn diagram of OTU numbers of abundant taxa across three periods (b) Venn diagram of OTU numbers of rare taxa across three periods

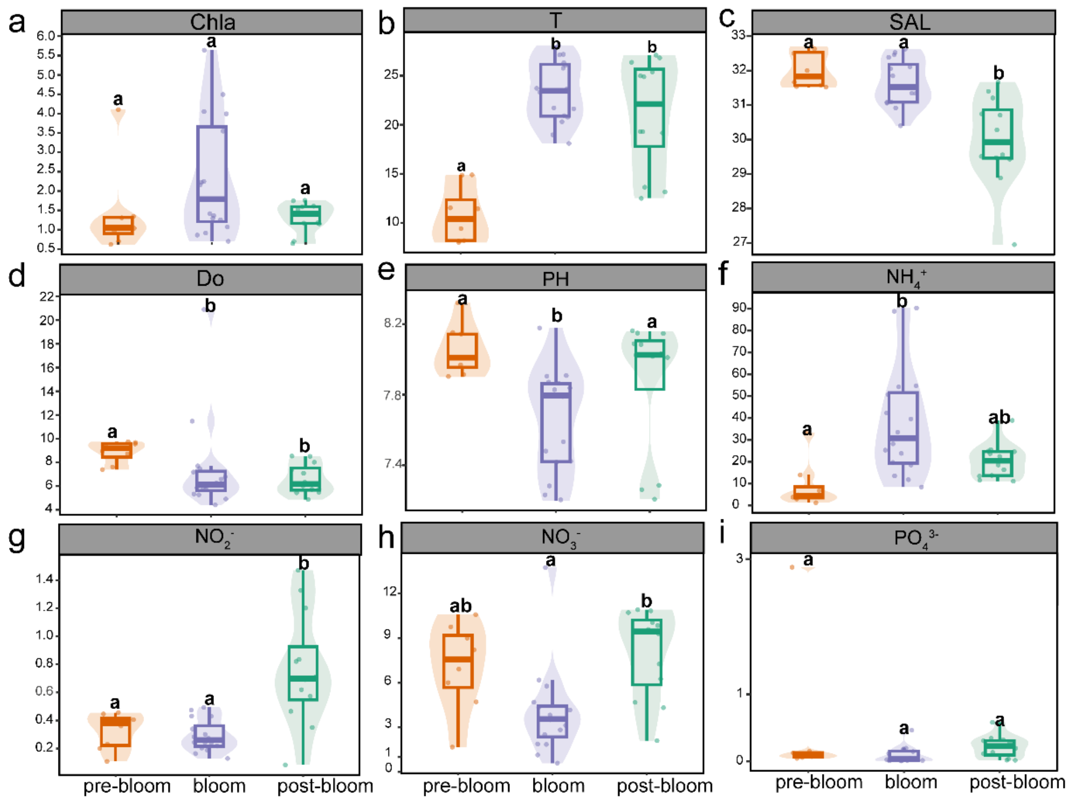

Figure S4. Box plot of environmental factor differences among three periods. (a) Chlorophyll a (Chl a); (b) Temperature (T); (c) Salinity; (d) Dissolved oxygen (DO); (e) PH; (f) ammonium (NH_4^+); (g) nitrite (NO_2^-); (h) nitrate (NO_3^-); (i) phosphate (PO_4^{3-}). Values with different lowercase letters are significantly different ($P < 0.05$).

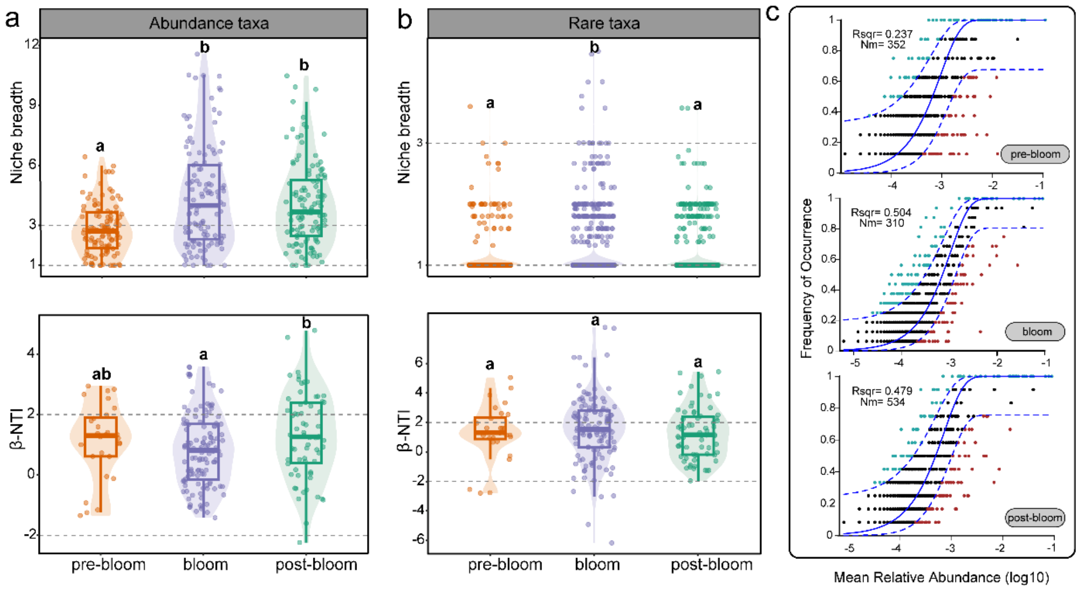

Figure S5. (a) Niche width and βNTI index of abundant taxa; (b) Niche width and βNTI index of rare taxa; (c) Neutral model analysis across three periods. Values with different lowercase letters are significantly different ($P < 0.05$).
